# Supplementary figures and images for: Effects of Different Photoperiods on the Transcriptome of the Ovary and Small White Follicles in Zhedong White Geese
Source: Animals (Basel). 2024 Sep 23;14(18):2747. doi: 10.3390/ani14182747 (PMC11428510; doi:10.3390/ani14182747)

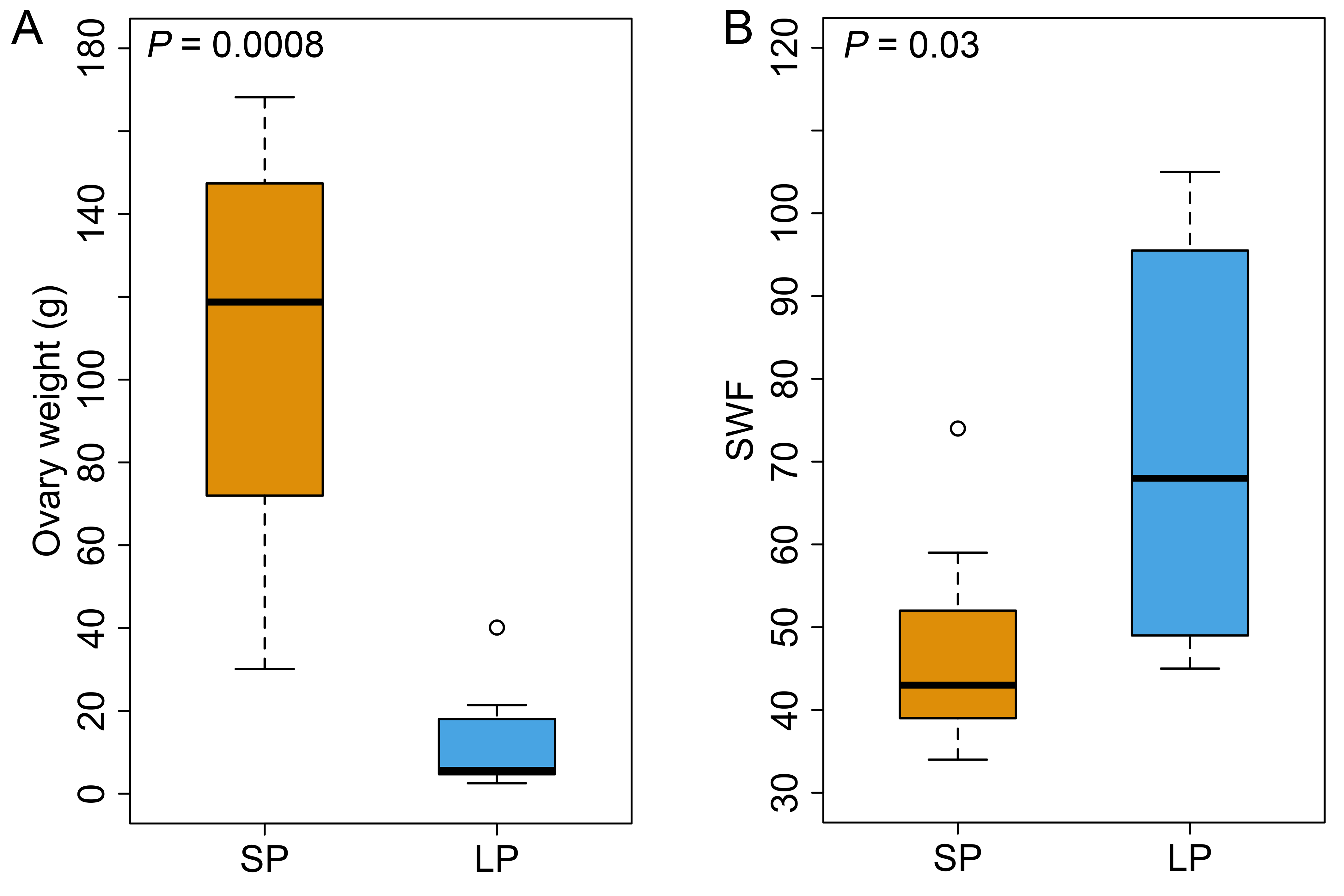

Supplement: Supplementary file 1 [file animals-14-02747-s001.zip › Figure S1.tif]

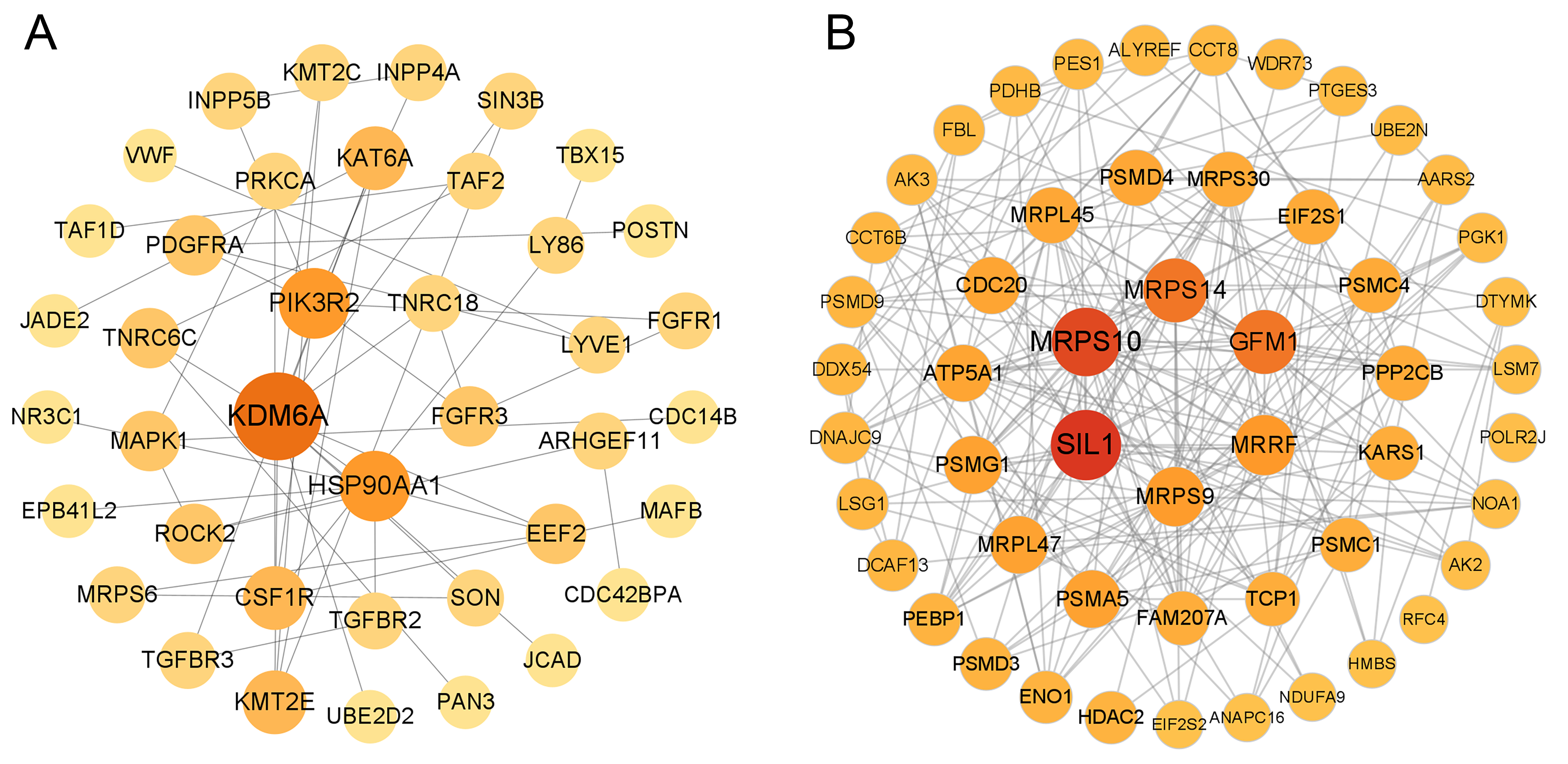

Supplement: Supplementary file 1 [file animals-14-02747-s001.zip › Figure S2.tif]

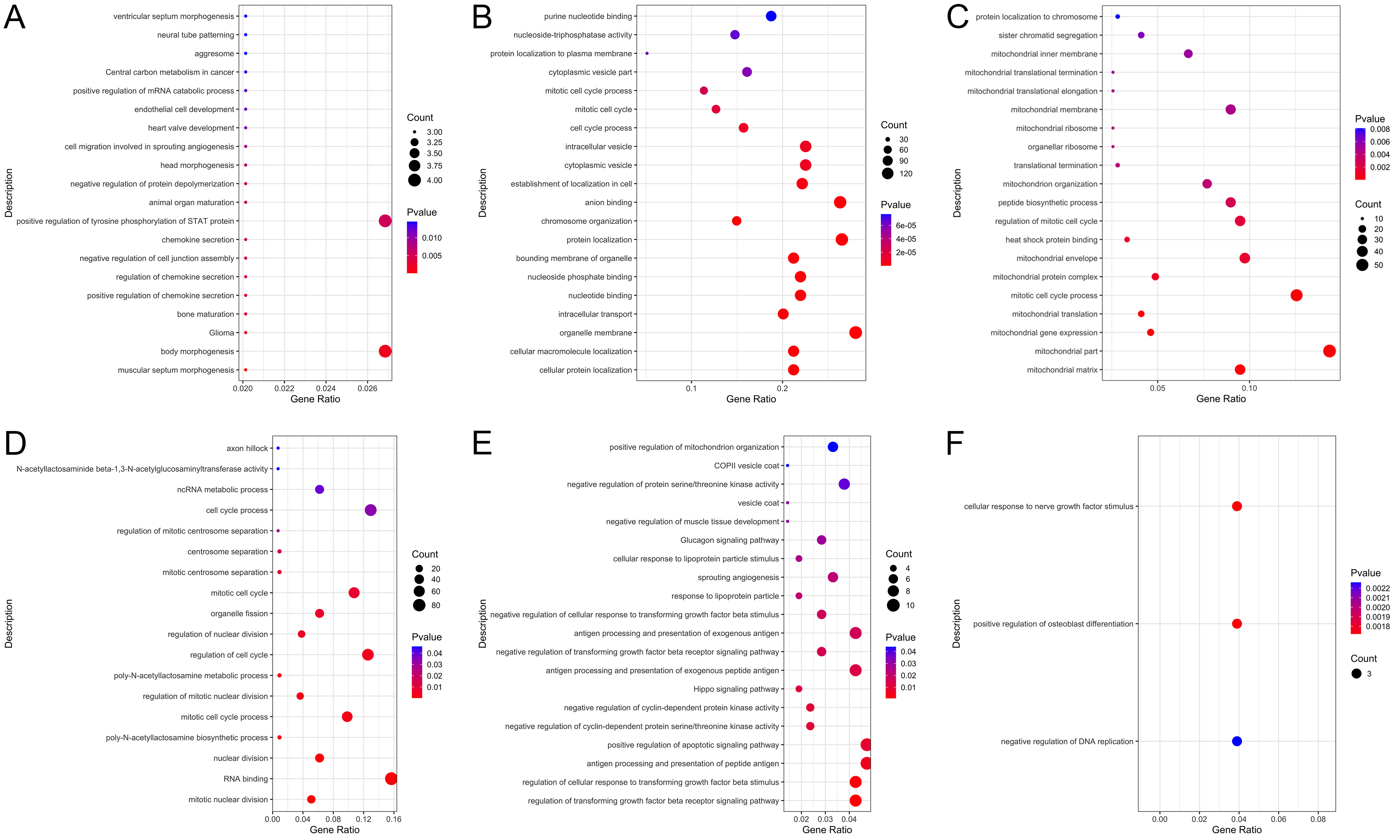

Supplement: Supplementary file 1 [file animals-14-02747-s001.zip › Figure S3.tif]
